# Sleep disturbances in orthopaedic trauma patients

**DOI:** 10.1097/OI9.0000000000000040

**Published:** 2019-07-09

**Authors:** Keyin Lu, John O. Barron, Heidi Israel, Lisa K. Cannada

**Affiliations:** a Saint Louis University School of Medicine; b Department of Orthopaedic Surgery, Saint Louis University, St. Louis, MO; c University of Florida-Jacksonville, Jacksonville, FL.

**Keywords:** fractures, ISI, orthopaedic trauma, PSQI, sleep disturbances

## Abstract

**Objectives:**

To determine the prevalence of sleep disturbances in orthopaedic trauma patients 3 months following surgery and to identify any subset(s) of patients at high risk for prolonged sleep disturbance.

**Design:**

Prospective cohort.

**Setting:**

Level 1 Trauma Center.

**Patient/Participants:**

All patients at an orthopaedic trauma clinic from May 3, 2016 to Feb 23, 2017.

**Main outcome measurements:**

Baseline patient Pittsburgh Sleep Questionnaire (PSQI) and Insomnia Severity Index (ISI) scores compared to PSQI and ISI scores 3-months postoperatively. Both gender and age stratified data analyses were performed.

**Results:**

Sixty-six patients met our inclusion criteria and completed both baseline and 3-month surveys. There were 44 males and 22 females. There was a significant increase in PSQI and ISI scores from baseline to 3 months across all patients. Further analysis revealed significant increases from baseline to 3 months in both PSQI and ISI scores for female patients but not male patients. There was a significant difference from baseline to 3 months in patients 50 years old and under but not for patients above 50 years old. No patients required revision surgery in the first 3 months.

**Conclusions:**

More than half of all patients reported continued sleep disturbance 3 months postoperatively. Females are at particularly increased risk for sleep disturbance. These findings demonstrate that sleep disturbances merit attention in the early stages of the postoperative recovery process.

**Level of evidence:**

Therapeutic Level II.

## 1 Introduction

Sleep represents an essential element for health and well-being, including physiological processes and quality of life, and the effects of sleep loss, both acute and chronic, have been well documented.^[1–4]^ Sleep disturbances are a common complaint among patients after orthopaedic trauma injuries, but they are often overlooked at follow-up visits. Furthermore, it can be unclear whether sleep disturbances were a problem prior to injury or if they are of acute onset. Recognizing sleep disturbances early during the postoperative period and managing such issues throughout long-term follow-up, especially in patients at risk for sleep disturbances, can help improve functional and emotional outcomes.^[5]^

It is well known that sleep disturbances pose a problem for patients immediately following surgery; however, whether they remain a problem throughout the surgery recovery process is not clear. Predictably, fatigued individuals have been shown to be less likely to adhere to home exercise regimens,^[6]^ implying that sleep throughout recovery may be particularly important if functional outcomes vary based on physical therapy adherence. This may be especially relevant as quality metrics that include patient satisfaction scores become increasingly important. The purpose of this study was to investigate the prevalence of sleep disturbances in orthopaedic trauma patients in the acute phase for 3 months following surgery and to identify any subset(s) of patients at increased risk for prolonged sleep disturbance.

## 2 Methods

### 2.1 Patient identification and enrollment

Patient enrollment was approved by the Institutional Review Board. Orthopaedic trauma patients at a Level 1 Trauma Center were screened at their first postoperative appointment, approximately 2 weeks following surgery. English-speaking patients age 18 to 75 years with an orthopaedic injury that required surgical stabilization were eligible for inclusion. Patients with a history of concussion within the last 6 months, brain surgery, stroke, those who reported significant pain for 3 or more nights per week prior to injury, or chronic narcotic medication use^[7]^ were excluded; such conditions can involve sleep disturbances for other, nonsurgical, reasons that we did not want to influence our findings. Once patients were screened and determined eligible for inclusion in the study, informed consent was obtained. Enrollment spanned from May 3, 2016 to February 23, 2017.

### 2.2 Measurement of sleep quality

Sleep quality was measured using two questionnaires, the PSQI and the ISI. The PSQI is a validated and internally reliable instrument that consists of 19 self-rated questions and 5 optional questions for a bed partner or roommate.^[8]^ The responses are totaled and scored, yielding using one global score. Global scores less than or equal to 5 indicate good sleep quality, and scores greater than 5 provide a sensitive and specific measurement of poor sleep quality relative to clinical and laboratory measures.^[8]^ The total score of 21 is derived from 7 domains of sleep: duration, disturbance, latency, dysfunction, efficiency, quality, and medication. The ISI is a validated and reliable instrument designed to measure perceived insomnia severity and to correspond with the DSM-IV's insomnia criteria.^[9]^ It consists of 7 self-rated questions that, like the PSQI, are summed to yield a global score. Scores 0–7 indicate no clinically significant insomnia, scores 8–14 indicate subthreshold insomnia, scores 15–21 indicate moderately severe clinical insomnia, and scores 22–28 indicate severe clinical insomnia. We received permission for use of both questionnaires.

### 2.3 Data collection

Two weeks following surgery, at their first postoperative appointment, eligible and consenting patients were asked to complete both the PSQI and ISI surveys based on their sleep the month prior to their injury; this established a baseline. Variables including patient age at the time of injury, sex, mechanism of injury, orthopaedic injury(s), nonorthopaedic injury(s), and surgery(s) were also recorded. The same patients were seen in clinic approximately 3 months later and asked to complete the PSQI and ISI again; this time based on their sleep from the time of their surgery. At 3 months, patients also answered questions regarding narcotic pain medication use, postoperative complications, utilization of assistive devices, return to work status, and use of tobacco, recreational drugs, or medications for sleep. The same 2 investigators performed all patient interviews.

### 2.4 Statistical analysis

Descriptive statistics, Chi-square, paired *t* tests, and Mann–Whitney nonparametric tests were utilized for analysis with SPSS 24.0 (IBM).

## 3 Results

### 3.1 Demographics

**Figure 1:**
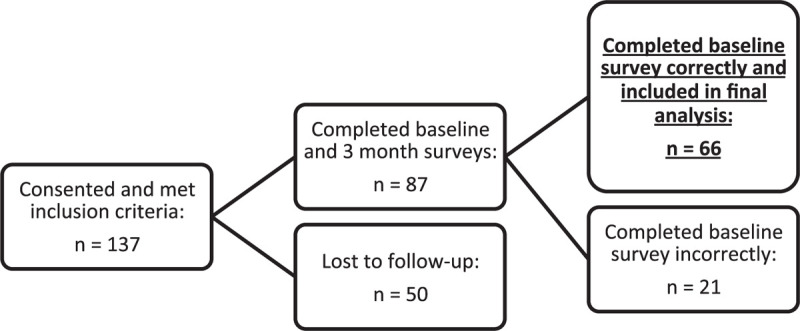
Patient enrollment and follow-up. One hundred thirty-seven patients consented to participate in the study after meeting inclusion criteria. Fifty patients were lost to follow-up, and every reasonable attempt was made to contact these patients. Eighty-seven patients completed both the baseline and 3-month surveys; out of these patients, 21 patients were later found to have filled out the baseline survey incorrectly.

One hundred thirty-seven patients initially agreed to participate in the study. Eighty-seven patients met our inclusion criteria and completed both baseline and 3-month PSQI and ISI surveys. Fifty patients who met our inclusion criteria were lost to follow-up and did not complete 3-month surveys. Upon final review of the survey responses, 21 patients were found to have completed their baseline or 3-month surveys incompletely or incorrectly and were therefore excluded from the final data analysis. This left 66 patients for final analysis (Fig. 1). There were 44 males and 22 females. The mean age was 44 years (range: 18–74). Forty-six (70%) of patients were under 50 years old.

### 3.2 Injury details

Twenty-eight patients (43%) were injured in a fall, 14 (21%) in motor vehicle accidents, 12 (18%) in motorcycle accidents, and 12 (18%) by other mechanisms of injury. Fall was the primary mechanism of injury for patients age 50 and over (75%) but was much less common for patients younger than 50 years old (28%). There were 12 patients with multiple fractures, 39 lower extremity fractures (hip, femur, tibia, foot/ankle, and periarticular), and 15 upper extremity fractures. No patients required revision surgery in the first 3 months.

### 3.3 Descriptive statistics

At the 3-month postoperative mark, 14 patients (21%) reported continued use of narcotic pain medications, and 8 (12%) continued to take over-the-counter (OTC) sleep aids. Of the 14 patients who reported continued narcotic use at 3 months, 12 were males and 2 females; thus 9% of female patients (2/22), compared to 27% (12/44) of male patients, continued to take narcotics at 3 months. Nine percent of female patients reported taking OTC sleep medication, compared with 14% of men. Twenty-five percent of patients less than 50 years old reported continued narcotic use at 3 months, compared to 20% of patients 50 years or older. Ten percent of patients less than 50 years old reported using OTC sleep medication at 3 months, compared to 13% of patients 50 years or older.

### 3.4 PSQI

**Figure 2:**
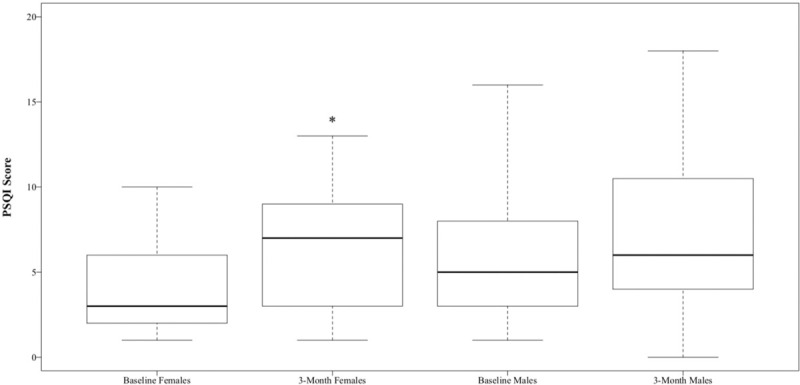
Continuous PSQI scores by sex. The horizontal line in the middle of each box indicates the median, while the top and bottom borders represent the 75th and 25th percentiles, respectively. The whiskers represent the minimum and maximum points. ∗ denotes statistically significant difference from baseline to 3 months for that population of patients.

**Figure 3:**
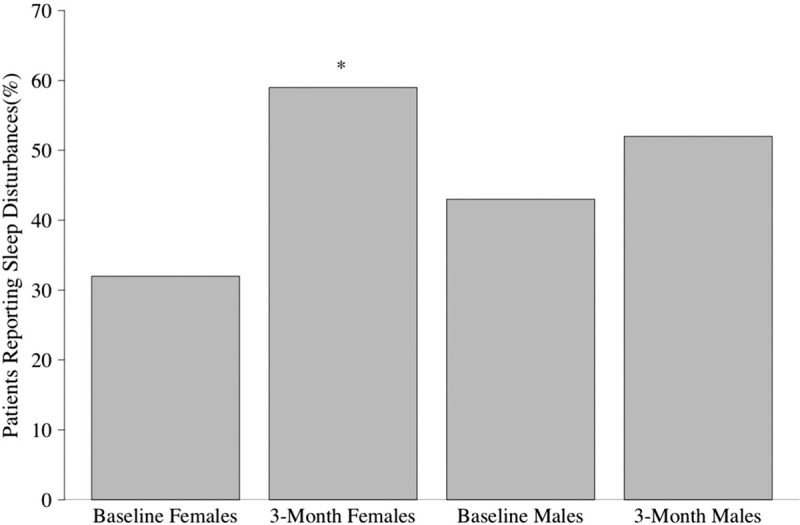
Categorical PSQI scores by sex. The bars represent the percentage and males and females that reported sleep disturbances (score >5) at both baseline and 3-months post-operatively. ∗ denotes statistically significant difference from baseline to 3 months for that population of patients.

Analysis of PSQI data revealed that at baseline, 39% of patients reported a sleep disturbance (PSQI > 5); this increased significantly to 56% 3-months postoperatively (*P* < .05, Table 1). Subsequent gender stratified analysis of both continuous and categorical PSQI data revealed significant differences in females but not males (Table 2). At baseline, 32% of females reported a sleep disturbance per PSQI, compared to 59% of females 3-months postoperatively (*P* < .05); 43% of males reported a baseline sleep disturbance per PSQI, compared to 53% of males 3 months postoperatively (Fig. 2, Table 1). There was also a significant increase in continuous PSQI scores in females from baseline to 3 months (*P* < .05), but there was not a significant increase in males (Fig. 3, Table 2). Patients under 50 years old also demonstrated a significant difference in baseline to 3-month scores (Table 2). Analysis of the PSQI subcategories that pertain to the 7 domains of sleep revealed significant differences in disturbance, latency, efficiency, and quality for all patients. Female patients showed the greatest decrease in sleep efficiency and sleep quality.

**Table 1:**
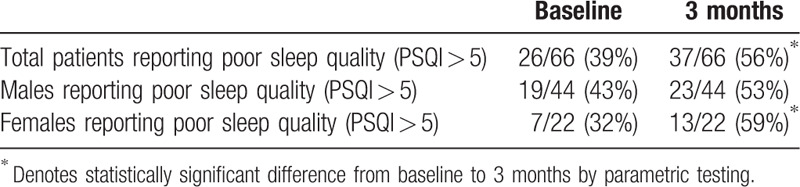
Number and percentage of patients reporting good and poor sleeping quality at baseline and 3 months.

**Table 2:**
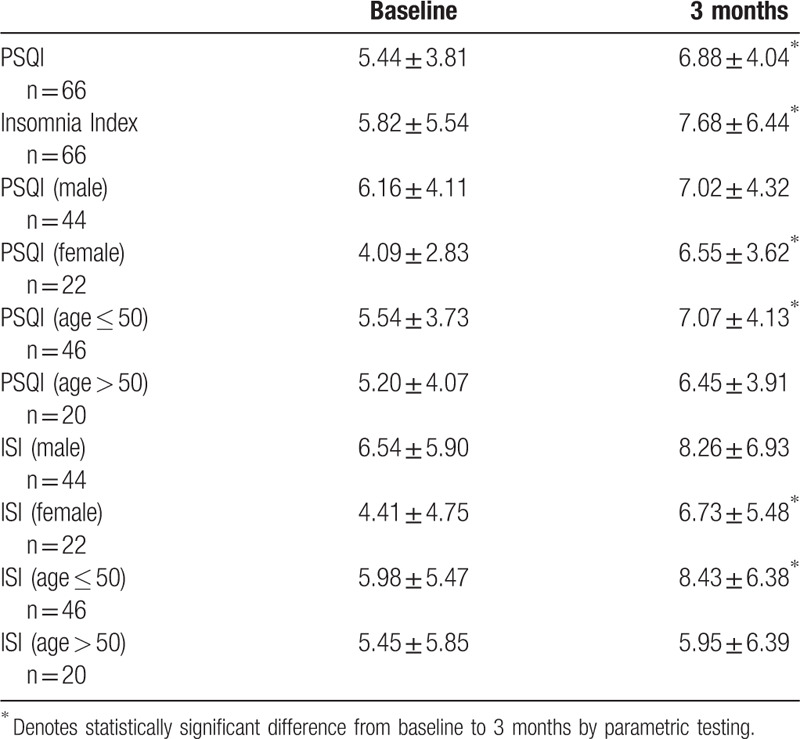
PSQI and ISI means with standard deviation at baseline and at 3 months.

### 3.5 Insomnia index

At baseline, 36% of patients reported at least some degree of insomnia (ISI > 7), and at 3 months, 45% of patients reported some degree of insomnia (Table 3). Analysis of continuous ISI data revealed a significant increase in ISI scores from baseline to 3 months, with a significant increase specifically in female patients and in patients younger than 50 years old (Table 2). There were no significant increases in male patients.

**Table 3:**
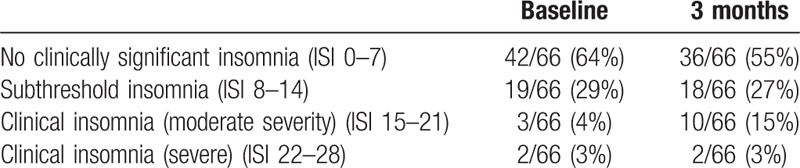
Number and percentages of patients reporting different levels of insomnia at baseline and 3 months.

## 4 Discussion

Sleep disturbances are known to be a common problem for patients immediately following surgery, and factors such as medications, postoperative pain, and the ICU environment are often cited as major causes.^[10–13]^ Various strategies, such as reduced opioid use and improved surgical techniques, have been implemented as part of efforts to reduce postoperative fatigue and improve convalescence.^[14]^ However, little research has been done to identify subsets of patients at particular risk for sleep disturbances. An understanding of the prevalence of sleep disturbances specifically following orthopaedic trauma surgery is vital for their identification and treatment in the orthopaedic population. Clinically, polysomnography represents the gold standard for sleep assessment; however, validated subjective surveys can provide a feasible option for large-scale measurement of sleep quality.^[15]^

The results indicated poor sleep quality was present 3 months after injury. Normally patients have healed fractures at that time and are progressing well in recovery. This finding emphasizes the impact of a traumatic event on patients which does not necessarily resolve as fractures heal. The majority of the trauma patients were male, but it was not unexpected to find an increase in sleep disturbances in females. These findings are consistent with previous studies on sleep quality in orthopaedic trauma patients and subsets of orthopaedic patients undergoing elective procedures.^[5,12,13,16]^ This being said, our findings suggest that sleep disturbances are a problem for orthopaedic trauma patients up to 3 months postoperatively. This knowledge provides a foundation for orthopaedic trauma surgeons to be more cognizant of the common nature of sleep disturbances in their patients, even months following surgery.

Females appear to be at increased risk for postoperative sleep disturbance compared to males, but at baseline, sleep disturbances were more prevalent in males. Interestingly, a similar pattern is seen when sleep disturbances are measured per PSQI in the general population.^[17]^ Furthermore, conditions such as posttraumatic stress disorder (PTSD) have been shown to be more prevalent in females and may provide a plausible explanation for the increased postoperative prevalence seen in females, given the traumatic nature of many of our patients’ injuries.^[18]^ We did not assess for PTSD and can only hypothesize that this may be a contributing factor. This hypothesis, however, is supported by Shulman et al,^[5]^ who found that at the 3-month mark, function has some relationship to poor sleep quality, but emotional difficulties have the greatest association with sleep quality in the long term. A systematic review by Smagula et al^[19]^ reported that female gender, poor physical health, and depressed mood were the most consistent independent predictors of sleep outcomes. Although we did not assess for depressed mood, our subset of patients who reported worsened sleep quality had 2 of the 3 independent factors identified in this study; the female patients, in a temporary state of poor physical health following their injury and surgery, were at higher risk for poor sleep outcomes.

Female scores were lowest in 2 domains of the PSQI, sleep efficiency and quality. Sleep efficiency is determined by the quotient of the number of hours of actual sleep by the number of total hours spent in bed. Sleep quality is determined by the patients’ rating of their overall sleep quality. Poor sleep efficiency can be seen with insomnia, defined as an inability to fall asleep or stay asleep. The first step to managing insomnia and poor sleep quality is to identify the underlying issue and recommend strategies for improved sleep hygiene. Some recommendations for improved sleep hygiene include avoidance of daytime naps, avoidance of caffeine late in the day, maintenance of a routine bed time, avoidance of prolonged screen time prior to bed, and reduction of distractions in the sleeping space, such as the TV or radio.^[20]^ Medications should be reviewed for potential iatrogenic causes of sleep disturbance. Objective quantification of the degree of sleep disturbance with a validated sleep questionnaire (PSQI and ISI) can be performed, even in busy clinical settings. Additionally, a tool such as the Patient Health Questionnaire-2 (PHQ-2) is a fast and effective way to screen for depression.^[21]^ Treatments should be aimed at the underlying cause of the sleep disturbance. For example, patients who meet criteria for depression, a common cause of sleep disturbance, may be referred for psychotherapy and/or started on a medication, such as a selective serotonin reuptake inhibitor, following further screening by their primary doctor. Those experiencing insomnia secondary to chronic pain should receive treatment focused on pain relief. Current guidelines recommend cognitive behavioral therapy as first-line treatment for chronic insomnia, while recommended pharmacotherapies include medications such as the non-benzodiazepine receptor agonists or melatonin receptor agonists such as ramelteon.^[22]^ Use of benzodiazepines, diphenhydramine, or melatonin is discouraged.^[22]^ More severe cases, or those refractory to initial interventions, may benefit from evaluation by a sleep specialist, who can investigate other potential etiologies, such as sleep-related breathing disorders or restless legs syndrome. Thus, orthopaedic physicians may help address their patients’ sleep disturbances by screening for common causes of sleep disturbance such as depression, recommending first-step solutions for improvement in sleep hygiene, and providing resources for further care when necessary.

Age-related differences were also seen; with younger patients affected more significantly. Shuman et al^[5]^ found that age was not a significant independent risk factor in sleep disturbances. The mechanism of injury may explain these findings, as younger patients tended to be injured in higher velocity motor vehicle accidents/motorcycle accidents crashes, and older patients tended to suffer falls. Because of the differences in injury mechanism between the 2 groups, it is difficult to attribute these findings to age alone.

Three months postoperatively, 27% of all males continued to take opioid medications. This was unexpected and somewhat disturbing, especially considering the increasing severity of the current opioid crisis. By contrast, a 2017 study by Brummett et al^[23]^ found that approximately 6% of opioid naive patients who undergo complex abdominal surgery report continued opioid use 3-months postoperatively. Although our sample size precluded analysis of the effects of continued opioid use on sleep, we felt that such a high percentage of continued opioid use was concerning and deserved attention. Further analysis of postoperative opioid use in orthopaedic trauma patient is warranted.

This study was limited in part by the design and population of patients. Postoperatively, patients were asked to recall their sleep patterns at their first office visit, when pain and sleep disturbance are both causing distress. Due to the accidental nature of orthopaedic trauma, it was not possible to collect baseline data prior to injury. We would have faced the same issue of potential recall error or bias had we enrolled patients at the time of admission for their traumatic injuries. This may have caused confusion for some patients when completing baseline surveys; however, extensive efforts were made to explain things to the patient and to answer any questions. Overall, baseline sleep disturbance prevalence per PSQI was 39%, just slightly higher than the 36% reported by Hinz et al^[17]^ in their recent PSQI analysis of 9284 members of the general population in Germany; suggesting that our patients did complete their baseline surveys in an unbiased manner, despite their recent injury and surgery.

We had a poor 3-month clinic follow-up rate, which may be explained by our patient population's broad geographical spread; however, this is purely anecdotal. We made every effort to contact patients if they missed their 3-month clinic appointment, and if possible, surveys were administered over the phone to reduce attrition bias. Despite this, we were ultimately unable to reach a larger than anticipated percentage of patients. The combination of some patients filling out baseline questionnaires incorrectly and a large percentage of patients lost to follow-up, meant we were unable to obtain 3-month data on approximately half of the patients initially enrolled, a significant limitation of the study.

Our sample size is relatively small, leaving potential for both type I and type II errors. Type I error is less likely, given that our baseline and 3-month sleep data match other studies. The chance of type II error is more likely, given that 43% of our male patients reported baseline sleep disturbances (PSQI > 5), while population studies report a baseline male sleep disturbance rate of 29%.^[17]^ Had baseline male scores been closer to the population average, a significant difference may have been seen. This variation in baseline male prevalence is likely due to our small sample size.

While the PSQI and ISI are validated and reliable instruments, they are subjective measures, and polysomnography remains the gold standard for diagnosing sleep disturbances.^[15]^ That being said, it could be argued that patient-reported outcomes are all the more important now. In an era where reimbursement can be tied to patient satisfaction, subjective, but validated and reliable surveys, are relevant tools for assessing and monitoring outcomes.

The strengths of our study were its prospective nature and the fact that we followed up with patients with an interview and data collection at 3 months. The same 2 investigators performed all patient interviews, providing continuity and ensuring consistency in data collection. This has not been replicated in the orthopaedic trauma literature.

## 5 Conclusions

In summary, sleep disturbance is a common complaint after orthopaedic trauma. More than 50% of patients reported continued sleep disturbance 3 months postoperatively. Females are at particularly increased risk for sleep disturbance, which we feel may be explained by psychological factors such as PTSD. These findings demonstrate that sleep disturbances merit attention throughout the entire postoperative recovery process. Such awareness can help ensure early postoperative detection of sleep disturbances so that both physical and emotional outcomes might be improved. Social support should be encouraged, and patients should be made aware of the commonness of sleep disturbances so that they may be better prepared to seek assistance should they be affected. Future research might seek to determine both the effect of sleep disturbance on functional outcomes and patient satisfaction, as well as the reason(s) for extended postoperative sleep disturbance. Lastly, given the current opioid crisis, it would be of benefit to specifically investigate the effect of long-term opioid use on postoperative sleep and outcomes.
